# Supplementary figures and images for: Tup1 is critical for transcriptional repression in Quiescence in S. cerevisiae
Source: PLoS Genet. 2022 Dec 21;18(12):e1010559. doi: 10.1371/journal.pgen.1010559 (PMC9815585; doi:10.1371/journal.pgen.1010559)

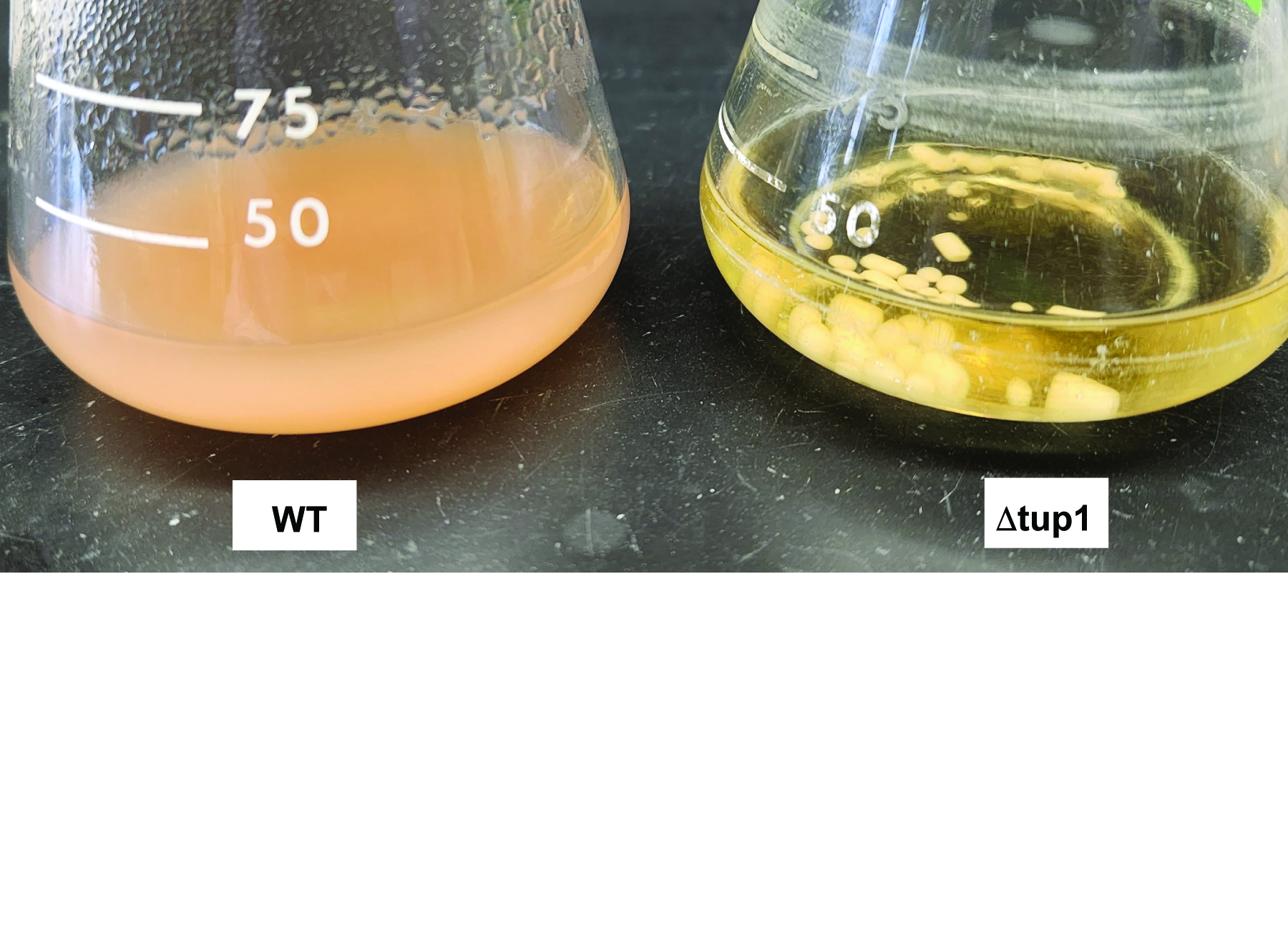

Supplement: S1 Fig — WT (left) and Δtup1 (right) yeast grown to log phase in YPD. (TIF) [file pgen.1010559.s001.tif]

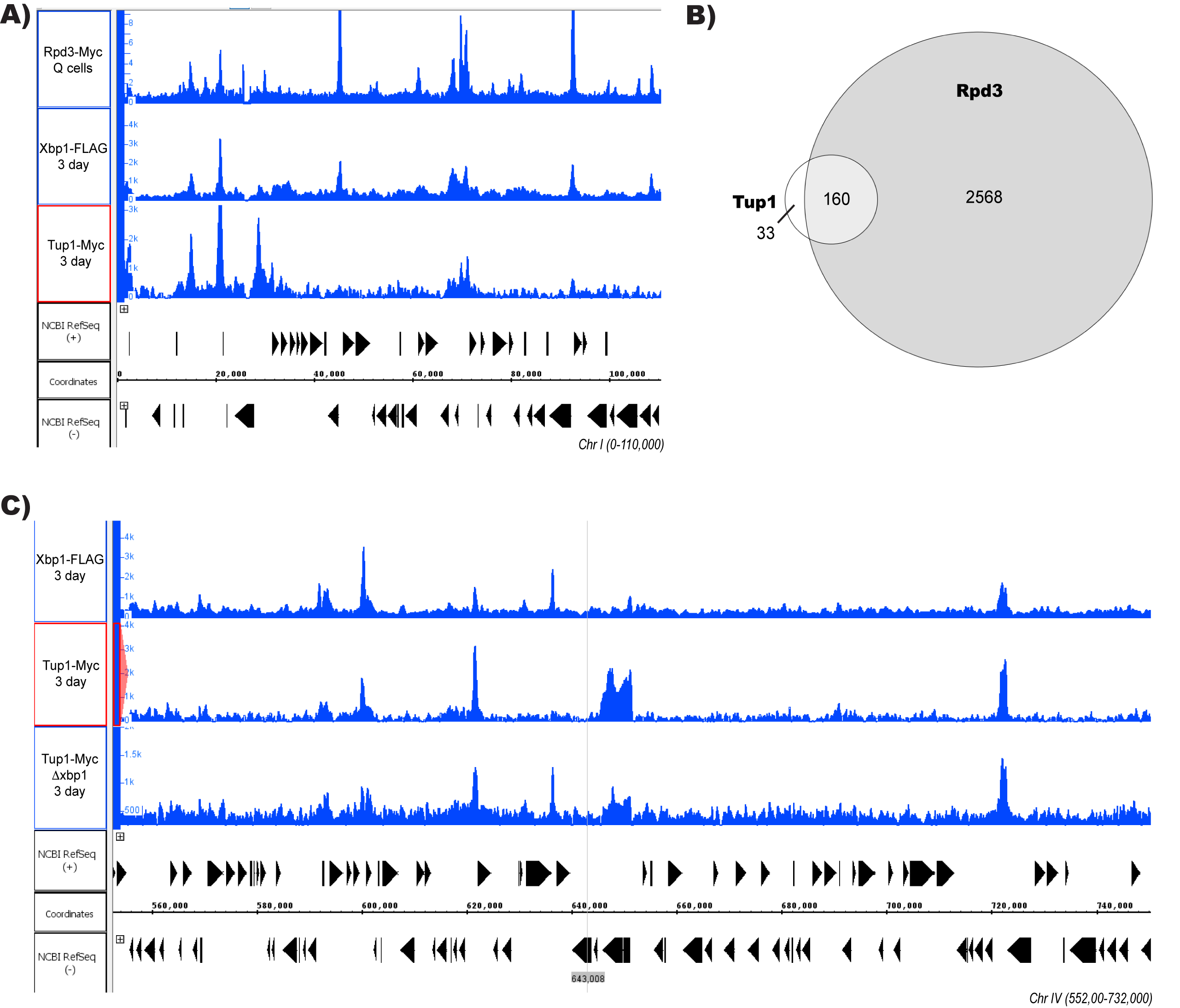

Supplement: S2 Fig — (A) Integrated Genome Browser (IGB) tracks representative of ChIP-Seq for Rpd3-Myc in quiescent cells (Q cells), Xbp1-FLAG in stationary phase (3 day), and Tup1-Myc in stationary phase (3 day). Rpd3-Myc data is from McKnight, Boerma et al. 2015. (B) Venn diagram comparing peaks from Rpd3-Myc ChIP of purified Q cells and Tup1-Myc ChIP of 3-day cultures. (C) Integrated Genome Browser (IGB) tracks representative of ChIP-Seq in stationary phase (3 day) for Xbp1-FLAG, Tup1-Myc, and Tup1-Myc in a Δxbp1 background. (TIF) [file pgen.1010559.s002.tif]
