# Supplementary material for: Tup1 is critical for transcriptional repression in Quiescence in S. cerevisiae
Source: PLoS Genet. 2022 Dec 21;18(12):e1010559. doi: 10.1371/journal.pgen.1010559 (PMC9815585; doi:10.1371/journal.pgen.1010559)
Supplement: S1 Table — TP = The number of primary sequences matching the motif / the number of primary sequences (the percentage of primary sequences matching the motif) TP% = The percentage of primary sequences matching the motif with scores greater than or equal to the optimal match score threshold FP = The number of control sequences matching the motif / the number of primary sequences (the percentage of control sequences matching the motif) FP% = The percentage of control sequences matching the motif with scores greater than or equal to the optimal match score threshold ENR_RATIO = The relative enrichment ration of the motif in the primary vs. control sequences, defined as Ratio = ((TP+1)/(NPOS+1)) / ((FP+1)/(NNEG+1)), where NPOS is the number of primary sequences in the input, and NNEG is the number of control sequences in the input SCORE_THR = The match score threshold giving the optimal p-value. This is the score threshold used by SEA to determine the values of “TP” and “FP” (PDF) [file pgen.1010559.s003.pdf]

ALT_ID	CONSENSUS	TP	TP%	FP	FP%	ENR_RATIO	SCORE_THR	PVALUE	LOG_PVALUE	EVALUE	LOG_EVALUE	QVALUE	LOG_QVALUE
Urc2p	HCGGAGWTA	57	29.23	12	6.15	4.46	13	3.330E-08	-17.22	0.0000243	-10.62	0.0000134	-11.22
Rap1p	ACACCCAYACAYYY	64	32.82	16	8.21	3.82	12	5.430E-08	-16.73	0.0000398	-10.13	0.0000134	-11.22
Tod6p	ADCGCGATGAGSTNHNV	28	14.36	1	0.51	14.5	13	7.890E-08	-16.36	0.0000577	-9.76	0.0000134	-11.22
Dot6p	ANGANGCGATGAGSTGH	24	12.31	0	0	25	16	8.090E-08	-16.33	0.0000592	-9.73	0.0000134	-11.22
Mot3p	TAGGTA	65	33.33	18	9.23	3.47	12	1.920E-07	-15.47	0.000141	-8.87	0.0000254	-10.58
Urc2p	CNTCGGAGATAWTT	57	29.23	15	7.69	3.63	12	5.610E-07	-14.39	0.000411	-7.8	0.0000619	-9.69
Met32p	ANTGTGGCGB	53	27.18	14	7.18	3.6	11	1.460E-06	-13.44	0.00107	-6.84	0.000138	-8.89
Rox1p	TCTATTGTTTCCC	54	27.69	15	7.69	3.44	8.8	2.160E-06	-13.05	0.00158	-6.45	0.000179	-8.63
Ste12p	TTGAAACAATGAAACG	50	25.64	13	6.67	3.64	8.4	2.430E-06	-12.93	0.00178	-6.33	0.000179	-8.63
Stp4p	DNNNNTTCAGCCGYACBAGNG	70	35.9	25	12.82	2.73	11	3.830E-06	-12.47	0.0028	-5.88	0.000253	-8.28
Tec1p	ACATTCTTACATTCTT	64	32.82	22	11.28	2.83	7.5	5.630E-06	-12.09	0.00412	-5.49	0.000339	-7.99
Azf1p	AAAAAGAAA	60	30.77	20	10.26	2.9	12	7.180E-06	-11.84	0.00526	-5.25	0.000396	-7.83
Matalpha2p	ATTGTT	76	38.97	30	15.38	2.48	8.9	8.230E-06	-11.71	0.00603	-5.11	0.000419	-7.78
YGR067C	GHGGGG	75	38.46	30	15.38	2.45	9.6	1.170E-05	-11.35	0.0086	-4.76	0.000556	-7.5
Upc2p	CTCGTWTAG	83	42.56	36	18.46	2.27	11	1.800E-05	-10.92	0.0132	-4.33	0.000795	-7.14
Srd1p	CNNTTTGTAGATCYCAH	72	36.92	29	14.87	2.43	10	1.960E-05	-10.84	0.0143	-4.25	0.000809	-7.12
Mot3p	WAGGTA	67	34.36	26	13.33	2.52	11	2.140E-05	-10.75	0.0157	-4.15	0.000835	-7.09
Pdr1p	TCCGCGGA	48	24.62	15	7.69	3.06	11	2.880E-05	-10.46	0.0211	-3.86	0.000953	-6.96
Pdr3p	TCCGCGGA	48	24.62	15	7.69	3.06	11	2.880E-05	-10.46	0.0211	-3.86	0.000953	-6.96
Rap1p	GGRTGTACGG	48	24.62	15	7.69	3.06	10	2.880E-05	-10.46	0.0211	-3.86	0.000953	-6.96
Ste12p	ATGAAACAATGAGACA	26	13.33	4	2.05	5.4	14	3.950E-05	-10.14	0.0289	-3.54	0.00115	-6.77
Msn1p	AATGTCCH	54	27.69	19	9.74	2.75	12	3.980E-05	-10.13	0.0291	-3.54	0.00115	-6.77
YKL222C	BAACGGARATAANNC	54	27.69	19	9.74	2.75	12	3.980E-05	-10.13	0.0291	-3.54	0.00115	-6.77
Tec1p	TTCTCACATTCTTC	71	36.41	30	15.38	2.32	9.1	4.720E-05	-9.96	0.0345	-3.37	0.00129	-6.65
Zap1p	TTCTTTATGGT	45	23.08	14	7.18	3.07	14	4.880E-05	-9.93	0.0357	-3.33	0.00129	-6.65
Hap1p	TAWCTCCG	55	28.21	20	10.26	2.67	12	5.110E-05	-9.88	0.0374	-3.29	0.0013	-6.64
Gat3p	AAATBRGATCTACAAGCTG	81	41.54	38	19.49	2.1	9.8	8.830E-05	-9.34	0.0646	-2.74	0.00216	-6.14
Tye7p	TGCRTCACGTGAYGCNNC	22	11.28	3	1.54	5.75	13	1.000E-04	-9.21	0.0732	-2.61	0.00236	-6.05
YML081W	CCCCDCH	107	54.87	57	29.23	1.86	10	1.110E-04	-9.1	0.0816	-2.51	0.00254	-5.97
Ste12p	CGTTTCA	68	34.87	30	15.38	2.23	13	1.280E-04	-8.96	0.0937	-2.37	0.00282	-5.87
Yrm1p	ACGGAAAT	66	33.85	29	14.87	2.23	12	1.510E-04	-8.8	0.11	-2.2	0.00322	-5.74
Yox1p	TWAATTR	29	14.87	7	3.59	3.75	7.4	2.080E-04	-8.48	0.152	-1.88	0.00423	-5.47
Arg81p	AAGTGCAACTGACTGCGA	25	12.82	5	2.56	4.33	7.6	2.110E-04	-8.47	0.154	-1.87	0.00423	-5.47
Sfl1p	THNHDNATAGAAGAAATAWDW	32	16.41	9	4.62	3.3	13	2.910E-04	-8.14	0.213	-1.55	0.00567	-5.17
Stp4p	TGCGCTABC	72	36.92	35	17.95	2.03	11	3.620E-04	-7.93	0.265	-1.33	0.00684	-4.99
Hcm1p	ATMAACAA	63	32.31	29	14.87	2.13	10	3.970E-04	-7.83	0.291	-1.24	0.00731	-4.92
Stp3p	GCTAGCGCA	55	28.21	24	12.31	2.24	12	4.800E-04	-7.64	0.351	-1.05	0.00859	-4.76
Lys14p	CNCAAAATTCCGBGCGNT	47	24.1	19	9.74	2.4	12	5.470E-04	-7.51	0.4	-0.92	0.00953	-4.65
Abf1p	RTCRYYY	51	26.15	22	11.28	2.26	10	6.690E-04	-7.31	0.49	-0.71	0.0114	-4.48
Fzf1p	YGSMNMCTATCAYTTYY	40	20.51	15	7.69	2.56	11	7.060E-04	-7.26	0.517	-0.66	0.0117	-4.45
Pdr8p	TCCGHGGA	88	45.13	49	25.13	1.78	9.2	9.120E-04	-7	0.668	-0.4	0.0147	-4.22
Rds2p	ANNNNGAAAAYCCGAGNNNNT	55	28.21	26	13.33	2.07	11	1.240E-03	-6.69	0.909	-0.1	0.0196	-3.93
Gsm1p	ANCTCCG	65	33.33	34	17.44	1.89	9.5	1.810E-03	-6.32	1.32	0.28	0.0262	-3.64
Mig1p	ATTTTGCGGGG	58	29.74	29	14.87	1.97	10	1.820E-03	-6.31	1.33	0.29	0.0262	-3.64

Mig2p	ATTTTGCGGGG	58	29.74	29	14.87	1.97	10	1.820E-03	-6.31	1.33	0.29	0.0262	-3.64
Mig3p	ATTTTGCGGGG	58	29.74	29	14.87	1.97	10	1.820E-03	-6.31	1.33	0.29	0.0262	-3.64
Gcr1p	CWTCC	52	26.67	25	12.82	2.04	9	2.000E-03	-6.21	1.46	0.38	0.0282	-3.57
Cep3p	YTCGGAAA	78	40	44	22.56	1.76	12	2.090E-03	-6.17	1.53	0.43	0.0288	-3.55
Cup2p	HTHNNGCTGD	27	13.85	9	4.62	2.8	12	2.490E-03	-5.99	1.83	0.6	0.0337	-3.39
Stb5p	CGGTGTTA	30	15.38	11	5.64	2.58	10	2.780E-03	-5.89	2.03	0.71	0.0368	-3.3
Rlm1p	CTAWWWWTAG	39	20	17	8.72	2.22	10	3.060E-03	-5.79	2.24	0.81	0.0397	-3.23
Tbf1p	ARCCCTAA	64	32.82	35	17.95	1.81	11	3.420E-03	-5.68	2.5	0.92	0.0435	-3.13
YKL222C	AACGGARAT	73	37.44	42	21.54	1.72	11	3.730E-03	-5.59	2.73	1.01	0.0466	-3.07
Arg80p	CCTCTAAAGG	51	26.15	26	13.33	1.93	9.2	4.100E-03	-5.5	3	1.1	0.0503	-2.99
Srd1p	GTAGWTC	42	21.54	20	10.26	2.05	9.6	4.800E-03	-5.34	3.51	1.26	0.0578	-2.85
Mcm1p	TTACCNAATTNGGTAA	110	56.41	72	36.92	1.52	4	4.960E-03	-5.31	3.63	1.29	0.0578	-2.85
Ixr1p	KTTSAAYKGTTYASA	105	53.85	68	34.87	1.54	6.6	4.970E-03	-5.3	3.64	1.29	0.0578	-2.85
Abf1p	AGCCGTAAATAGTTATCTTCCAAG	33	16.92	14	7.18	2.27	0.15	5.130E-03	-5.27	3.76	1.32	0.0586	-2.84
Aft2p	CGCACCC	101	51.79	66	33.85	1.52	9.4	6.670E-03	-5.01	4.88	1.59	0.0749	-2.59
Yap1p	ASGTAATT	108	55.38	72	36.92	1.49	10	7.240E-03	-4.93	5.3	1.67	0.0799	-2.53
Nrg2p	HAGGGTCB	50	25.64	27	13.85	1.82	11	7.970E-03	-4.83	5.83	1.76	0.0865	-2.45
Cad1p	TTACGTAAT	152	77.95	109	55.9	1.39	7.5	8.230E-03	-4.8	6.02	1.8	0.0879	-2.43
Pdr3p	VCGGAA	47	24.1	25	12.82	1.85	8.8	8.600E-03	-4.76	6.3	1.84	0.0894	-2.41
Dal81p	GAAAATTGCGTT	55	28.21	31	15.9	1.75	10	8.780E-03	-4.74	6.43	1.86	0.0894	-2.41
Dal82p	GAAAATTGCGTT	55	28.21	31	15.9	1.75	10	8.780E-03	-4.74	6.43	1.86	0.0894	-2.41
Met32p	TGTGGCDB	132	67.69	93	47.69	1.41	8.9	9.450E-03	-4.66	6.92	1.93	0.0948	-2.36
Rds1p	CCBBTCGGCCGAAVDNCD	89	45.64	58	29.74	1.53	7.2	1.000E-02	-4.61	7.32	1.99	0.0988	-2.31
Rlm1p	TAWWWWTAGM	81	41.54	52	26.67	1.55	9.2	1.110E-02	-4.51	8.09	2.09	0.103	-2.27
Met32p	ABTGTGGC	67	34.36	41	21.03	1.62	10	1.120E-02	-4.49	8.22	2.11	0.103	-2.27
Mig1p	TTATTTCTGGGG	108	55.38	74	37.95	1.45	6.4	1.130E-02	-4.48	8.28	2.11	0.103	-2.27
Mig2p	TTATTTCTGGGG	108	55.38	74	37.95	1.45	6.4	1.130E-02	-4.48	8.28	2.11	0.103	-2.27
Mig3p	TTATTTCTGGGG	108	55.38	74	37.95	1.45	6.4	1.130E-02	-4.48	8.28	2.11	0.103	-2.27
Mot3p	AAGGWT	50	25.64	28	14.36	1.76	11	1.140E-02	-4.48	8.32	2.12	0.103	-2.27
Msn1p	AATGTCC	39	20	20	10.26	1.9	12	1.190E-02	-4.43	8.69	2.16	0.106	-2.24
Stb5p	CGGKGTTATA	73	37.44	46	23.59	1.57	9.9	1.210E-02	-4.41	8.89	2.19	0.107	-2.23
Rdr1p	TNNGNCNTGCGGAWATNNNNC	60	30.77	36	18.46	1.65	12	1.280E-02	-4.36	9.39	2.24	0.111	-2.2
Rph1p	AANNNDAWTTAGGGGKGNA	84	43.08	55	28.21	1.52	9.5	1.290E-02	-4.35	9.41	2.24	0.111	-2.2

Supplementary Table 1: Motif Analysis of Tup1 ChIP in Stationary Phase (3 day)

TP = The number of **primary** sequences matching the motif / the number of primary sequences (the percentage of primary sequences matching the motif)

TP% = The percentage of primary sequences matching the motif with scores greater than or equal to the optimal match score threshold

FP = The number of **control** sequences matching the motif / the number of primary sequences (the percentage of control sequences matching the motif)

FP% = The percentage of control sequences matching the motif with scores greater than or equal to the optimal match score threshold

ENR_RATIO = The relative enrichment ration of the motif in the primary vs. control sequences, defined as

Ratio = ((TP+1)/(NPOS+1)) / ((FP+1)/(NNEG+1)),

where NPOS is the number of primary sequences in the input, and NNEG is the number of control sequences in the input

SCORE_THR = The match score threshold giving the optimal *p*-value. This is the score threshold used by SEA to determine the values of “TP” and “FP”
